# Adsorption of Glycine on TiO_2_ in Water from On-the-fly Free-Energy Calculations and In Situ Electrochemical Impedance Spectroscopy

**DOI:** 10.1021/acs.langmuir.4c00604

**Published:** 2024-05-21

**Authors:** Lorenzo Agosta, Luca Fiore, Noemi Colozza, Guillermo Pérez-Ropero, Alexander Lyubartsev, Fabiana Arduini, Kersti Hermansson

**Affiliations:** † Department of Chemistry-Ångström Laboratory, Uppsala University, Uppsala 751 21, Sweden; ‡ Department of Science and Chemical Technologies, University of Rome Tor Vergata, Via della Ricerca Scientifica, Rome 00133, Italy; § Department of Chemistry-BMC, Uppsala University, Ridgeview Instruments AB, Uppsala 752 37, Sweden; ∥ Department of Materials and Environmental Chemistry, Stockholm University, Stockholm 106 91, Sweden

## Abstract

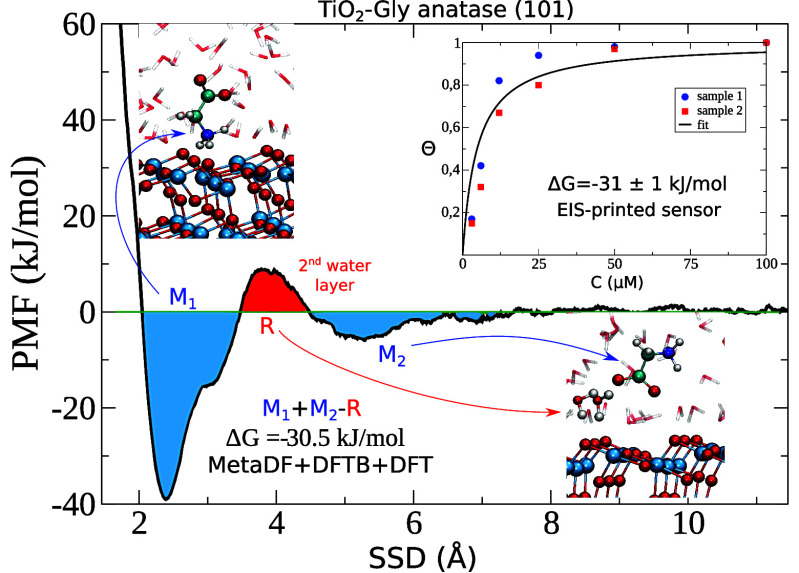

We report here an experimental-computational study of hydrated TiO_2_ anatase nanoparticles interacting with glycine, where we obtain quantitative agreement of the measured adsorption free energies. Ab initio simulations are performed within the tight binding and density functional theory in combination with enhanced free-energy sampling techniques, which exploit the thermodynamic integration of the unbiased mean forces collected on-the-fly along the molecular dynamics trajectories. The experiments adopt a new and efficient setup for electrochemical impedance spectroscopy measurements based on portable screen-printed gold electrodes, which allows fast and in situ signal assessment. The measured adsorption free energy is −30 kJ/mol (both from experiment and calculation), with preferential interaction of the charged NH_3_^+^ group which strongly adsorbs on the TiO_2_ bridging oxygens. This highlights the importance of the terminal amino groups in the adsorption mechanism of amino acids on hydrated metal oxides. The excellent agreement between computation and experiment for this amino acid opens the doors to the exploration of the interaction free energies for other moderately complex bionano systems.

## Introduction

Specific molecular recognition by inorganic materials^1,2^ is a central concept in nanodrug delivery^3^ and for nanotoxic responses,^4^ as well as in many other applications where the penetrative and adhesive properties of adsorbing (bio)molecules onto an inorganic surface play a role.^4−7^ Among inorganic surfaces, nanostructured metal oxides are particularly interesting substrates due to their high concentration of surface-active sites and, for some applications, also their redox and photocatalytic powers.^8^ Nanostructured TiO_2_, for example, is utilized in medicine^9,10^ for its high biocompatibility. In biological fluids, metal oxide nanoparticles (NPs) are coated with a corona of proteins that determines the cellular response.^11−13^ This corona links to the NPs only by a few small peptides, but the factors that govern the peptide-NP affinity are much debated and mostly unknown. For instance, it has been observed that the surface lattice spacing at the exposed NP facets can have a significant influence on the adsorption mechanism,^14−16^ while surface defects have been suggested to have minor impact.^17^ Moreover, the configurational entropy, the specific amino acid sequence, and their side chains play fundamental roles in the peptide adsorption process.^18−20^

Deciphering the respective contributions to the free energy of adsorption from the side chains and terminal groups is key to understanding the adsorption mechanism. A significant step forward in this direction was accomplished by phage display^1,18,21^ and NMR^22^ techniques, which demonstrated how charged amino acids drive the adsorption of small peptides on TiO_2_. In particular, it was shown that arginine, lysine, as well as the positively charged amino terminating groups of neutral amino acids contribute more to the peptide adsorption than negatively charged counterparts like aspartate, glutamate, and the carboxylate-terminating groups of the neutral amino acids. Similarly, the adsorption free energy of small peptides were extracted by means of quartz crystal microbalance measurements by Sultan et al.,^20^ who identified glycine, lysine, and arginine as being the leading adsorbing amino acids, whereas aspartate was reported to give no contribution. Nevertheless, the problem of quantifying the contribution of single amino acid side chains to the total peptide adsorption free energy remains mostly unknown as the interaction of the amino and carboxyl terminal groups with the surface can make significant contributions.

In the present study, we address bioadsorption on a metal-oxide surface in bulk water, namely, the adsorption free energy of glycine (Gly) on hydrated titanium dioxide (TiO_2_, anatase) NPs. Glycine has been studied for its polymerization properties on metal/metal-oxide surfaces.^14,17^ It is a zwitterion in aqueous solution (at pH 7) under ambient conditions and the only amino acid that does not possess a side chain. Thus, its interaction with the environment is driven merely by the terminal amino and carboxyl groups, making it a perfect candidate to single out the biointeraction of the terminal groups with the solid surface. Knowing the contribution of the terminal groups to the total adsorption interaction of a peptide (or even of a single amino acid) is one essential key to understanding their bioadhesion mechanism onto metal oxides.

In addition to the challenges of deciphering different contributions to the adsorption free energy, there is also the challenge of determining its total value. For example, Sano et al.^23^ estimated the adsorption free energy of the RKLPDA peptide using phage display and reported a value of ≈−30 kJ/mol, while Suzuki et al.,^22^ using NMR, reported a value of ≈−12 kJ/mol for the same peptide. On the other hand, free-energy measurements of isolated amino acid molecular adsorption at TiO_2_–water interfaces have only rarely been investigated. At neutral pH, Langmuir models fitted on (i) HPLC adsorption data^24^ have given values ranging from −8 to −20 kJ/mol for all the 21 amino acids, even for the nonpolar amino acids and (ii) infrared spectroscopy measurements of lysine^25^ gave a free-energy value of −15 kJ/mol, excluding direct binding of the charged side chain to the TiO_2_ surface. These studies show that the terminal NH_3_^+^ and COO^–^ group contributions play a considerable role in the adsorption process, which impedes capturing the real side-chain surface affinity.

In this paper, we evaluate the adsorption free energy of glycine on a fully hydrated anatase TiO_2_ surface, both computationally and experimentally. On the computational side, we perform molecular dynamics (MD) simulations that retain the electrons, i.e., we perform ab initio MD (AIMD) simulations, thereby allowing the description of polarization, reactivity, and charge-transfer effects. Many classical MD simulations (i.e., with force-fields, no electrons present) combined with free-energy calculations have been published in the literature for amino acids on wet TiO_2_ surfaces, reporting a very wide range of adsorption free-energy values for charge amino acids and their analogues.^26−34^ We also note that many classical pair potentials tend to overestimate the water–TiO_2_ interactions (see discussions in ref (35) and references therein), which likely implies overstructured interfacial water, resulting in artificially hinder adsorption and diminish the adsorption free energy. Furthermore, the adhesion of polar molecules on TiO_2_ promotes polarization effects that are generally not described in classical force fields and therefore would require specific and targeted reparametrization.^36^ Limitations such as these, entailing classical force fields, highlight the merits of using electronic structure methods, as we do here. However, free-energy calculations based on electronic structure calculations generally require an unfeasible amount of computational time. In this paper, however, we combine metadynamics techniques with thermodynamics integration and speed up the convergence of the free-energy calculation by at least a factor 100 compared to standard metadynamics.^37^ To the best of our knowledge, the current paper is the first attempt to sample the glycine adsorption free-energy landscape by electronic methods.

Also the experimental investigations in our paper adopt a new approach: we sample the Langmuir isotherm by electrochemical impedance spectroscopy (EIS) on screen-printed sensors. Compared to most techniques used in the literature, our method adopts a quick, cheap, and portable setup (with a user-friendly interface). Our results presented in this article show, possibly for one of the first time, very good agreement between the experimental and computational free-energy evaluations for glycine on titania and provide insights about the binding modes (one in contact with the surface, one solvent-mediated). Furthermore, our study demonstrates the importance of having an advanced description of the water adsorbed at the interface by keeping the electronic information both in our MD and in our metadynamics simulations. We find that the water molecules mediate the surface’s interaction with the NH_3_^+^ and COO^–^ groups, the former being the main responsible for the adsorption in the presence of liquid water.

## Materials and Methods

Our computational work involves two types of quantum-mechanical electronic structure methods, namely, density functional tight-binding (“DFTB”),^38,39^ and the more advanced density functional theory (“DFT”), and two types of statistical-mechanical methods for the exploration of the phase space and the free-energy landscape, namely, MD simulations and Metadynamics (“MetaD”) simulations. Here, we perform the MetaD simulations with the efficient approach “MetaDF”^37^ alluded to in the Introduction (the “F” refers to force integration). Altogether, thus, we make use of four types of methodologies: “DFTB-MD”,“DFT-MD” (or AIMD as it is often called), “DFTB-MetaDF”, and “DFT-MetaDF”. In addition, single-point DFT and DFTB calculations were performed for validation of the adsorption energies (see as listed in Table S1 in Supporting Information for further details). All the computations were performed with CP2K software^40^ coupled with PLUMED.^41^

Concerning our description of the interface system under study, we use here a pristine TiO_2_ anatase slab exposing the (101) surface, which has been calculated to be the most thermodynamically stable facet, and thus largely exposed at the NP interface (see e.g. ref (42)). The (001) facet, although also present in anatase NPs, represents a considerable minor amount of the total exposed surface due to its instability and water-induced reconstruction.^43−45^ Moreover, all water molecules in our system are intact. We did not observe any dissociation during our AIMD or DFTB-MD simulation run. This is also in agreement with our previous AIMD results.^46^ Indeed, although water dissociation has been found experimentally at low water coverage,^47^ it has not been shown that it persists at room temperature or in the presence of liquid water, as was pointed out in a second harmonic generation study of this surface under aqueous conditions^48^ and a recent ab initio MD study.^49^

The experimental approach used here relies on the interaction of the glycine molecules adsorbed on the TiO_2_ NPs deposited on a gold sensor surface, producing a adsorbate–TiO_2_ complex on the working electrode. We used a TiO_2_ nanopowder, mainly composed of anatase phase.^24,50^ The adsorption of glycine on TiO_2_ anatase was monitored using EIS, a technique that can probe charge changes at the surface electrodes with high sensitivity and accuracy. By measuring the charge-transfer resistance (*R*_ct_)^51,52^ of a redox process occurring between a solution of 5 mM ferro-ferricyanide (containing 0.1 M KCl) and the electrode surface, it was possible to evaluate the amount of molecules adsorbed on the TiO_2_ powder (see the Supporting Information for further details). We note that our experimental approach is operated in situ as it consists of a portable easy-to-use printed sensor. It allows fast analysis (<1 min for each glycine concentration measured by scree-printed electrodes), and it avoids the use of organic solvents, replaced by the use of only a few microliters of aqueous solutions for each analysis.

## Results and Discussion

The adsorption on the TiO_2_ anatase (101) surface relies on the possibility for glycine to penetrate the layered water structure naturally forming at the very interface.^28,31,37,53^ Indeed, in aqueous environment, where most bioapplications operate, water molecules adsorb onto the under-coordinated metal and oxygen atoms at the surface and form a layer-like structure,^8,54^ which mediates the interactions with the adsorbing molecules.^55^ The structure of this solid–liquid interface, including its protonation state, regulates the adhesion of molecules, and it is thus fundamental for understanding the adsorption mechanism.^7,8,56^

**Figure 1:**
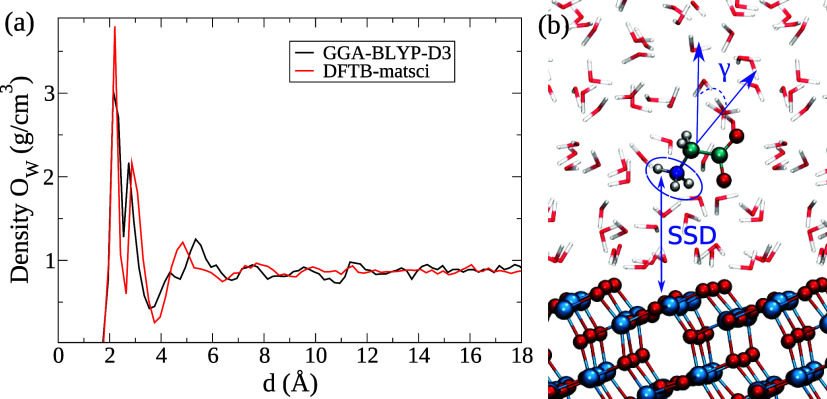
(a) Water oxygen density profiles for the MD simulations using TB-Matsci parameters and with DFT-GGA approach. The small differences between the first peaks in the profiles can be attributed to the difference in box size. (b) Representation of the hydrated Gly-anatase (101) system and the collective variables SSD and γ for the Metadynamics simulation. The text sometimes refers to the first, second, and third water layers, which corresponds to the peak order in the density profile, and to the related dominant structural motifs, namely (in order), water molecules oriented with O_w_ toward the surface, water molecules orienting at least one OH bond toward the surface, and waters in the 4–6 Å region. Several examples are seen in this figure and in Figure 2.

In Figure 1a (black curve), the density of the water oxygen atoms along the surface normal is reported for the DFTB-MD simulation containing only the TiO_2_ slab and water molecules (without Gly). The *x*-axis (*d*) denotes the distance between the water oxygen atoms and the outermost Ti atoms. For comparison, the water oxygen density profile of a hydrated anatase (101) surface simulated with the DFT-MD (BLYP-D3) method, taken from ref (46), is also reported (red curve). It is evident that the two water structures at the interface are quite similar and that the DFTB Matsci parameters appear to reproduce the DFT-GGA features.

We can identify three water layers: water molecules adsorbed on the five-coordinated titanium surface atoms, Ti_[5]_, giving a density peak at *d* ≈ 2.3 Å, water hydrogen bonded to the bridging surface oxygens, O_br_, at *d* ≈ 3.0 Å, and a third-layer of water molecules at *d* ≈ 5.0 Å. The modest differences between the two methods, especially for the first peak in the density profile, can be accounted for by the different surface lattice spacing, resulting from small differences in cell parameters used to create the respective supercells (see Table S1 in Supporting Information).

The potential of the mean force (PMF) was calculated by spanning the free-energy landscape with two collective variables: the distance of the NH_3_^+^ group from the TiO_2_ slab (SSD) and the an internal angle of glycine (see Figure 1 and Supporting Information for more details). Thus, with the MetaDF method applied to our system, the Gaussians history^31,57^ of the bias potential is combined with thermodynamic integration of the mean forces acting on the NH_3_^+^ atoms along the collective variable SSD (see Figure 1b)
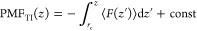
1where *z* spans the SSD-values from *r*_c_ (the onset of the solid surface to 1 nm where the potential wall is set). ⟨*F*(*z*)⟩ is the canonical averaging of the average force weighted on the 2D bias potential.^37^ Combining metadynamics with thermodynamic force integration was demonstrated to provide a fast convergence for the PMF.^58^ Here, the unbiased atomic forces acting on the SSD collective variable are extracted “on-the-fly” along the MetaDF simulations, before the correction added by the bias potential (contrary to the method of Marinova and Salvalaglio^58^ where the mean force is recalculated from the added bias potential). This allows us to obtain incremental averaged forces every single MD step and to reach a converged PFM profile in about 200–300 ps of simulation per walker.^37^

The final adsorption free energy is computed as
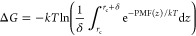
2where *k*_B_*T* is the product of the Boltzmann constant and the absolute temperature, δ is the thickness of the adsorption layer, and *r*_c_ + δ indicates the beginning of the liquid bulk.

**Figure 2:**
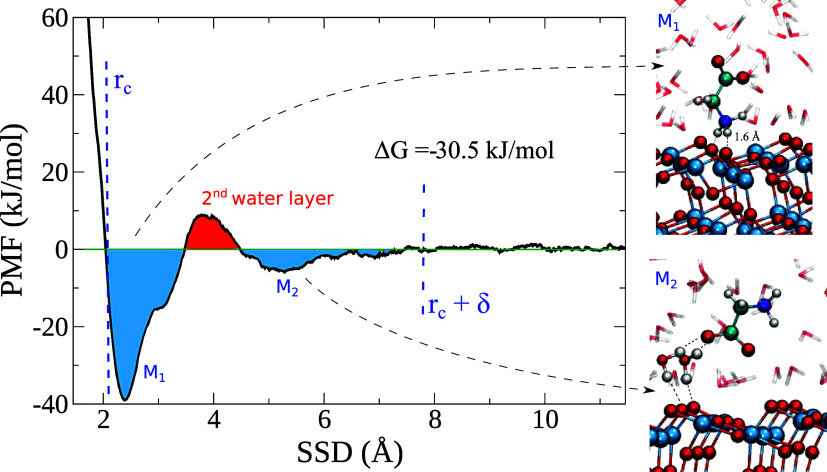
PMF of Gly adsorbing on the TiO_2_ anatase (101) surface. Two adsorption modes can be identified *M*_1_ and *M*_2_ separated by an energy barrier of 5 kJ/mol. *M*_1_ represents the adsorption via the NH_3_^+^ group to the O_br_ atoms while *M*_2_ occurs by adsorption to the second water layer. The total adsorption free energy is computed by eq 2 using the illustrated *r*_c_ and δ values.

The PMF calculated with the MetaDF method along the SSD variable is reported in Figure 2 showing a converged adsorption profile after 300 ps (per walker) of simulation. Two main adsorption modes can be observed. The first one, *M*_1_, consists of a direct bidentate interaction of the NH_3_^+^ group with two O_br_ atoms which have lost their adsorbed waters. The amino group has been reported in ab initio studies to be able to interact either via O_br_ or via the second water layer above the titania surface.^37,59,60^ The free-energy valley spans a range of 1.5 Å, which approximately corresponds to the first and second water peaks in Figure 1a.

The adsorption free energy calculated through eq 2 with δ = 1.5 for this mode is ≈−27 kJ/mol. The NH_3_^+^ group must replace the water molecules adsorbed on the O_br_ sites, and this requires overcoming a free-energy barrier of ≈5 kJ/mol (red area in Figure 2). This barrier lies within the second and third water layers (Figure 1a), a region which has been reported to possess restrained water dynamics,^53^ which might impede the glycine mobility at the interface. The NH_3_^+^ free-energy value agrees with previous classical computational studies,^30,32−34^ although they reported lower adsorption free energies (between −12 and −30 kJ/mol) and experimental work,^20,22,25^ indicating the charged amino group to be responsible for binding the TiO_2_ surface.

The mean (H···O_br_) distance of the N–H···O_br_ hydrogen bond is ≈1.6 Å, which indicates the formation of a rather strong hydrogen bond. We calculated the adsorption energy of the *M*_1_ mode in the absence of water using both the DFTB and DFT approaches, as mentioned in the Materials and Methods section. The resulting *E*_ads_ values are 120 ± 3 kJ/mol with both methods. The bidentate adsorption mode implies an adsorption energy of 60 kJ/mol per N–H···O_br_ bond, which is quite large to be ascribed to a N–H·· hydrogen bond between neutral species, but here, the H-bond acceptors are oxide ions (albeit partly screened).

**Figure 3:**
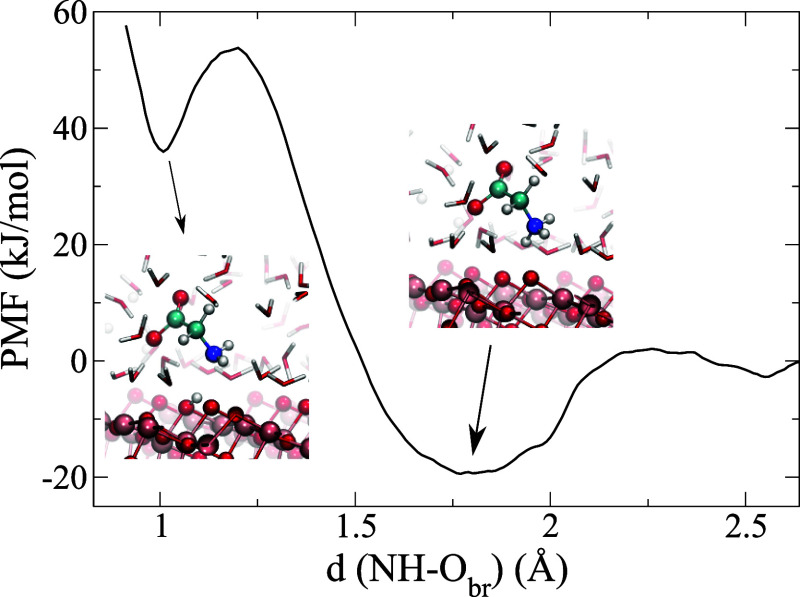
PMF of the deprotonation process from a positive charged NH_3_^+^ group to the O_br_ site on the anatase (101) surface.

In order to further validate the large free energy of the *M*_1_ mode, we computed the free-energy value needed for a hydrogen of the NH_3_^+^ group to deprotonate on the neighboring O_br_ (see the Computational Method section). We adopted in this case the DFT-MetaDF approach in order to avoid the tight-binding restraints. The result reported in Figure 3 illustrates that an hydrogen needs to overcome a barrier of 50 kJ/mol in order to be adsorbed on the TiO_2_ surface, forming a shallow metastable state of 5 kJ/mol. We also note that the energy for the NH_3_^+^ group to move away from the surface is about −14 kJ/mol per hydrogen atom involved in the adsorption. This is in good agreement with the DFTB-MetaDF calculation, confirming the validity of the approximations taken in this model.

We now turn to the second adsorption mode, *M*_2_. Classical MD studies have reported that the COO^–^ group adsorbs onto the Ti_[5]_ site on the TiO_2_ rutile surface for glycine^28^ and acidic amino acids such as aspartate and glutamic acid^32,34^ while adsorption via the second water layer was found from ab initio simulations for acidic amino acids on anatase.^37,59^ For the *M*_2_ adsorption mode in the present study, the carboxylate group accepts hydrogen bonds from water molecules adsorbed on the bridging surface oxygen and residing in the second TiO_2_ hydration shell. A relatively small adsorption free energy of ≈−8 kJ/mol was calculated for this mode. We did not observe any direct adsorption of the COO^–^ group onto the Ti_[5]_ sites. The low adsorption free-energy value and the rotational mobility of water molecules linked to the O_br_ sites prevent us from identifying a unique adsorption configuration for the *M*_2_ mode (see Supporting Information). We note that this mode might be more dominant by lowering the pH of the solution, as reported in IR adsorption studies.^61,62^ Overall the low adsorption free energy for this mode is in line with the observation that negatively charged amino acids, at neutral pH, do not guide the peptide adhesion on oxide surfaces in the presence of water^15,16,20,22,25,63,64^ (while it is the contrary for dry conditions).

In summary, our metadynamics simulations show that for glycine, the total adsorption free energy is −30.5 kJ/mol (within a statistical error of 1 kJ/mol), and it consists of the sum of the two adsorption modes *M*_1_ and *M*_2_ minus the free energy barrier between them.

We also performed a MetaDF simulation considering glycine in its negatively charged state. This resulted in a null value of the adsorption free energy (see Figure S2), which confirms that the anchoring group in glycine is NH_3_^+^.

In order to validate the adsorption energy value obtained by our simulations, we estimated the same quantity by constructing a Langmuir isotherm for glycine adsorbing on TiO_2_ by means of EIS (see Materials and Methods and Supporting Information). Increasing concentrations of glycine (i.e., from 1 to 300 μM) were analyzed on the gold sensor coated with the TiO_2_ powder, by washing the electrode surface with distilled water after each measurement. This range of concentration is a factor 1000 lower than the concentration needed for polymerization of glycine^14,17^ on TiO_2_, enabling discard gly–gly coupling effects upon adsorption. Our study was conducted by using two independent electrodes. All the measurements were carried out in the absence of UV light in order to avoid photocatalytic activation of TiO_2_. We note that the adsorption of Gly on the bare gold electrode yielded an *R*_ct_ value that did not vary as a function of the glycine concentration. Therefore, we could discard the possibility that the electrode had affected the glycine adsorption process on TiO_2_ NPs.

The adsorption free energy of glycine on TiO_2_ was evaluated by assuming that *R*_ct_ is proportional to the amount of molecules adsorbed on the TiO_2_ NPs deposited on the gold electrodes. Under this assumption, it is possible to build a Langmuir isotherm where the *R*_ct_ data are fitted to the standard Langmuir equation^65^
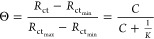
3where Θ is a function of the charge-transfer resistance *R*_ct_ (which is proportional to the molecule surface coverage), and it was calculated from two different independent replicas. Θ is scaled to vary in the range of 0–1, which represents the number of occupied adsorption sites over the total amount of adsorption sites per surface area.  is the value of the bare gold electrode covered with TiO_2_, and  is the asymptotic value of *R*_ct_. *R*_ct_ values were obtained by ZView software, selecting the fitting curve having the lowest error associated with the *R*_ct_. *C* is the molar concentration of the molecules, and *K* is the equilibrium constant for the adsorption. Once *K* is known, the adsorption free energy can easily be derived from
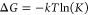
4

**Figure 4:**
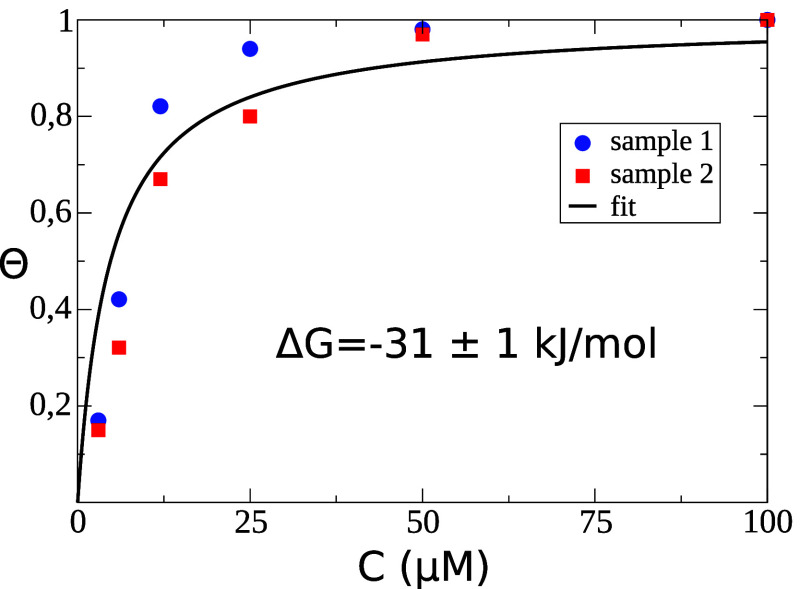
Adsorption Langmuir isotherm for glycine adsorbing on the TiO_2_ nanopowder. The fitted curve is obtain as the best fit of two different independent replicas (see Supporting Information for the *R*_ct_ values).

The *R*_ct_ values extracted from Nyquist plots for different glycine concentrations (see Supporting Information) are used to fit a Langmuir isotherm using eq 3. The results are reported in Figure 4 for two independent replicas. The data match a Langmuir behavior very well, indicating that the Gly adsorption occurs on the TiO_2_ NPs surface, as expected.

The experimentally derived adsorption free energy according to eqs 3 and 4 is −31 ± 1 kJ/mol for glycine. This Δ*G* value is in excellent agreement with the MetaDF calculations.

This is the first time that experimental and computational results for the adsorption free energy of single amino acids on a metal oxide surface have been compared with such consistent results, as far as we are aware. These results not only lend credibility to the techniques we have used but also offer interesting possibilities for future investigations of the adsorption of small biomolecules on solid–liquid interfaces.

## Conclusions

We combined experimental and computational techniques in order to assess the adsorption thermodynamics of glycine on the TiO_2_ anatase (101) surface. Dynamic electronic structure calculations were performed to sample the adsorption free-energy landscape. The metadynamics simulations made use of a recent development which couples the metadynamics framework with thermodynamics integration of the unbiased mean force collected on-the-fly along the MD trajectories. This allows a speed-up of the calculations’ convergence which enabled us to perform free-energy simulations that explicitly include the electrons in every dynamic step for this complex system. Including the electrons allowed us to describe the water structure at the interface with polarization and charge-transfer effects taken into account. On the experimental side, we introduced a new and quick method to construct a Langmuir isotherm from in situ EIS measurements on a screen-printed sensor from which accurate estimate of the adsorption free energy could be obtained.

The results show that the second hydration layer on the TiO_2_ surface constitutes a moderate barrier for the glycine adhesion, which anyway strongly interacts with the bridging oxygens through the charged amino group NH_3_^+^. The carboxylate group, instead, is responsible for weak adsorption on the second TiO_2_ hydration shell and cannot adsorb on the Ti_[5]_ sites. The negatively charged glycine is not able to adsorb on the surface, confirming the importance of the charge amino group, and in general of the terminal amino-carboxylate group, in amino acid adhesion on metal oxides. The overall adsorption free energy is estimated to be about −30 kJ/mol for glycine, in excellent agreement with the experimental value extracted from the EIS measurements. The large adsorption energy is due to strong hydrogen bonding between the charged NH_3_^+^ group and the TiO_2_ bridging oxygens, which highlights the importance of considering ab initio accuracy to tackle the bioadsorption on metal oxides. The methodologies presented in this paper can readily be extended to other biomolecules and NPs for a systematic investigation of biointeractions on solid–liquid interfaces.

## Supporting Information Available

All technical details about the simulation and experimental methods; 2D map of the CVs used in the MetaD simulation of Gly; PMF and walker phase sampling for the case of negatively charged Gly; and typical Nyquist plot from which the *R*_ct_ values have been derived (PDF)
