# Supplementary material for: Adsorption of Glycine on TiO2 in Water from On-the-fly Free-Energy Calculations and In Situ Electrochemical Impedance Spectroscopy
Source: Langmuir. 2024 May 21;40(23):12009–16. doi: 10.1021/acs.langmuir.4c00604 (PMC11171457; doi:10.1021/acs.langmuir.4c00604)
Supplement: Supplementary file 1 — la4c00604_si_001.pdf [file la4c00604_si_001.pdf]

# Supporting Information: Adsorption of Glycine on $TiO_2$ in water from on-the-fly free energy calculations and in-situ electrochemical impedance spectroscopy


Lorenzo Agosta,[*,†] Luca Fiore,[*,‡] Noemi Colozza,[‡] Guillermo Pérez-Ropero,[¶] Alexander Lyubartsev,[§] Fabiana Arduini,[‡] and Kersti Hermansson[†]

†*Department of Chemistry-Ångström Laboratory, Uppsala University, 751 21 Uppsala, Sweden*

‡*Department of Science and Chemical Technologies, University of Rome Tor Vergata, Via della Ricerca Scientifica, 00133 Rome, Italy*

¶*Department of Chemistry-BMC, Uppsala University, Ridgeview Instruments AB, 752 37 Uppsala, Sweden,*

§*Department of Materials and Environmental Chemistry, Stockholm University, 106 91 Stockholm, Sweden*

E-mail: lorenzo.agosta@kemi.uu.se; luca.fiore@uniroma2.it

## Computational method

For DFTB, the Self Consistent Charge Density Functional Tight-Binding approach[1] was used with the Matsci parametrization[2] for the atomic pair interactions. No long range corrections were used. The DFT calculations were performed with the GGA-BLYP functional[3,4] and the Grimme D3[5] dispersion correction. All simulations were performed with the CP2K[6] code, coupled with the PLUMED software[7] for Metadynamics.

**System preparation.** The simulation boxes were prepared with the PACKMOL[8] software. The box sizes were fixed at values as reported in the right-most column of Table S1. A $TiO_2$ anatase (101) slab containing $4 \times 3 \times 3$ unit cells repetitions was used while the remaining space in the simulation box was filled with one glycine molecule and water at its relative density for 1 atm and 310 K (see Fig. 1b in the main text).[9] The systems were set to the temperature of 310 K by rescaling the velocities with the thermostat of Bussi[10] and using an integration time step of 0.5 fs. A DFTB-MD simulation was run for 50 ps for a system containing only the $TiO_2$ anatase (101) and water in order to evaluate the water structure at the interface for the Matsci parameters. The structures were compared with those from DFT-MD simulations taken from Ref.[9] where the GGA-BLYP functional[3,4] with the Grimme D3[5] dispersion correction were used.

Table S1: Simulations details. All simulations were run here except the DFT-MD entry, where the trajectory was taken from Ref.[9]

| **Method** | **Code** | **System** | **Simulation run and box size (Å$^3$)** |
|---|---|---|---|
| DFTB-MD (Matsci) | CP2K | $TiO_2$-water | 50 ps, $10.35 \times 11.08 \times 40.00$ |
| DFT-MD (BLYP-D3) | CP2K | $TiO_2$-water | 50 ps, $10.55 \times 11.40 \times 43.00$ |
| DFTB-MetaDF (Matsci) | CP2K-PLUMED | $TiO_2$-water-Gly | 2.6 ns, $10.55 \times 11.40 \times 43.00$ |
| | | $TiO_2$-water-$(Gly)^-$ | 2.6 ns, $10.55 \times 11.40 \times 43.00$ |
| DFT-MetaDF(BLYP-D3) | CP2K-PLUMED | $TiO_2$-water-Gly | 10 ps, $10.55 \times 11.40 \times 43.00$ |

**Enhanced sampling.** Metadynamics simulations were run within the well tempered diffusive approach[11–13] with a bias factor of 15. Gaussians with an initial height of 3.5 kJ/mol were added every 25 fs and the widths were updated every 75 ps. These parameters were demonstrated to constitute a good compromise for ab-initio Metadynamics simulations, where the accessible sampling is strongly limited in time.[14] In order to speed up the phase space sampling we made use of 8 walkers[15] starting from different initial configurations. A potential wall at 1 nm away from the outermost row of Ti atoms was introduced to restrict the sampled phase space. Each walker was run interchanging information with the remaining walkers every 25 fs. The Glycine side chain consists of a single hydrogen atom and at physiological pH the amino-carboxylate groups carry fractional local charges, usually denoted $NH_3^+$ and $COO^-$ for simplicity. This is also the natural state predicted from

ab-initio DFT simulations. The Matsci parameters make glycine be stable in its neutral non-zwitterionic form.

In order to maintain the amphoteric state we imposed a constraint on the NH and CH bond length to avoid spurious deprotonation.[14] The Matsci parameters were constructed for water-titania interfaces[2] but it is known that bulk water is not well described (the water-water interactions are underestimated). However, the free energy evaluation in MetaDF is based on the mean force acting on the adsorbing molecule. In the bulk region, the water density is uniform and isotropic, and thus Gly will feel an overall null mean force acting on it, which does not affect the evaluation of the free energy according to Eq. 1 in the main text.

We also checked the effect of these constraints on the adsorption free energy by performing the same simulation with a glycine molecule in its negatively charged form (using $COO^-$ and $NH_2$ as terminal groups, as shown in Fig. S2.

The DFTB-MetaDF simulations were run considering two independent collective variables for sampling the adsorption free energy landscape. The surface separation distance (SSD) variable is defined as the distance between the outermost layer of Ti atoms on the $TiO_2$ surface and the center of mass of the $NH_3{}^+$ group in glycine (Gly) amino acid (see Fig. 1b in the main text). Using the $NH_3{}^+$ group instead of the center of the mass of the whole molecule allows us to directly assess the adsorption modes.[14] The second variable was defined as the angle ($\gamma$) between the normal vector to the $TiO_2$ surface and the vector defined by the distance between $N-C_N$ in Gly molecule ($C_N$ is the first carbon neighbour atom to the Gly nitrogen, Fig. 1b in the main text). This variable was used in order to enhance the sampling of the phase space and it was integrated out in the calculation of the free energy. Up to 320 ps were simulated for each walker.

Moreover, a DFT-MetaDF simulation was run using a single walker on the collective variable measuring the distance between an H atom of the $NH_3{}^+$ group and the neighbouring $O_{br}$ site. Those results are given in connection with the deprotonation discussion and Fig. 3 in the main text.

**Static single-point DFT and DFTB calculations.** As electronic structure method validation, we also performed single-point calculations to compare the adsorption energy from DFT and DFTB for the most stable glycine binding mode on the dry $TiO_2$ surface. The adsorption energy $E_{ads}$ of the molecule on the slab was calculated as $E_{ads} = E_{slab+mol} - E_{slab} - E_{mol}$ where $E_{sup}$ and $E_{gly}$ are the energies of two separated systems composed of the bare anatase (101) slab and the isolated Gly molecule in the same position as in the adsorption mode that corresponds to $E_{slab+mol}$. Here the DFT and DFTB electronic structure methods were the same as those used in Table S1, i.e. the Matsci parametrization for DFTB, and BLYP-D3 for DFT.

### Experimental section

Commercial $TiO_2$ nanopowder (Sigma-Aldrich 718467; $\geq$99.5%) was used to coat the screen-printed electrodes. This powder is composed of particles of 21 nm, a typical size for which the anatase phase is stable and exposes the (101) facet.[16,17] The adsorption of glycine on $TiO_2$ anatase was monitored using the electrochemical impedance spectroscopy (EIS), a technique that can probe charge changes at the surface electrodes with high sensitivity and accuracy.

By measuring the charge transfer resistance ($R_{ct}$) of a redox process occurring between a solution of 5 mM ferro-ferricyanide (containing 0.1 M KCl) and the electrode surface it was possible to evaluate the amount of molecules adsorbed on the $TiO_2$ powder. Herein, the Randles equivalent circuit[18] was used as a model for the electronic components of our sensor, integrating a Constant Phase Element in the circuit to take account of the double layer capacitance. A sinusoidal potential was applied to the electrochemical cell scanning a wide range of frequencies ($\Omega$). Subsequently measurements of the resulting impedance ($Z(\omega)$) were obtained. The $R_{ct}$ values were determined from a Nyquist plot,[19] which is composed of the imaginary part of ($-Z''$) plotted as a function of the real part ($Z'$). The extrapolation of the semicircles onto the x-axis of the Nyquist plot yields the $R_{ct}$ value, obtained with the ZView® software (see Supp. Info).

Gold screen-printed electrodes (SPEs, S4M-PE08G) from Sense4Med company were used for the EIS experiments that were carried out with a portable potentiostat PalmSens3 (PalmSens Instrument), connected to a laptop equipped with PsTrace software.

For the EIS measurements, the gold electrodes were modified by sequential drop-casting of 4 µL of a $TiO_2$ dispersion ($TiO_2$ 1 mg/mL) in distilled water, for a total amount of 12 µL. The modified sensors were allowed to dry on a hot plate at 100 °C. Measurements were carried out in the range from 10000 Hz to 1 Hz, with an increase of 10 points per frequency decade, thus scanning 41 frequencies. A potential of 0 V was applied to the electrochemical cell, with a potential amplitude of 0.01 V.

## S1. 2D map of Glycine collective variables

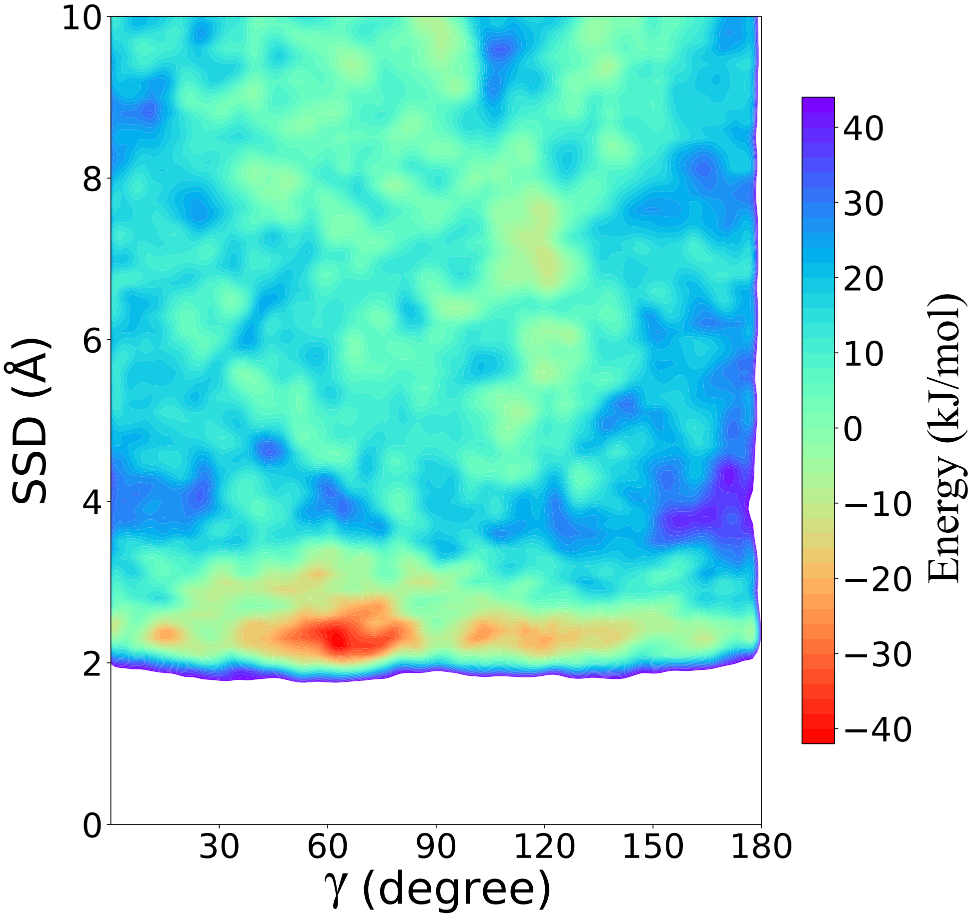


Figure S1: 2D plot of the accumulated bias potential for the SSD and $\gamma$ collective variables describing the Gly adsorbing on hydrated $TiO_2$ anatase (101) surface.

In Fig. S1, the 2D accumulated bias potential is plotted for the SSD and $\gamma$ collective variables. It is well visible that the $M_1$ mode has a preferential orientation of about $60^o$ with respect to the surface normal. This is due to the bidentate adsorption of two hydrogen atoms on the $O_{br}$ atoms and it indicates a strongly reduced mobility with respect to the bulk solution. The $M_2$ mode does not display a preferential adsorption orientation due to the fact that it occurs on the second water layer, where there are no orientational constraints.

**S2. PMF and walkers dynamics for the negative charged Gly**

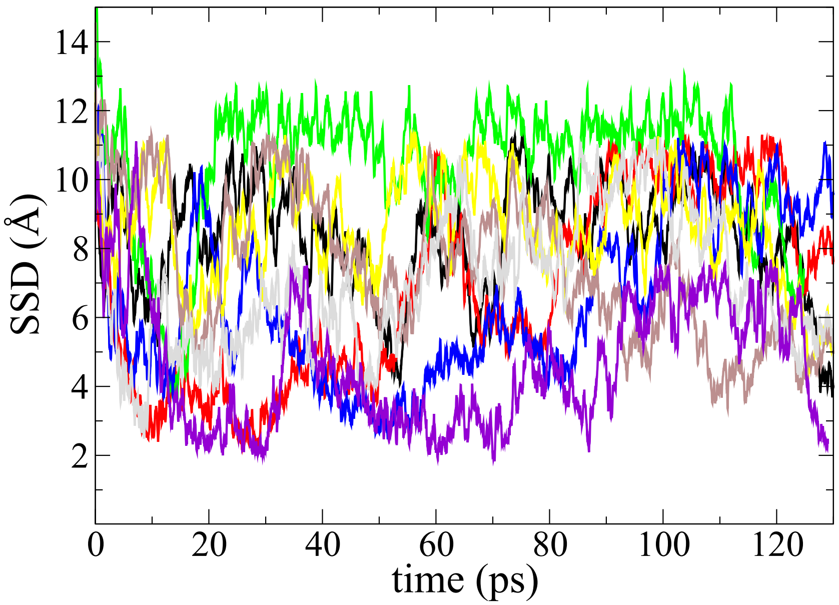


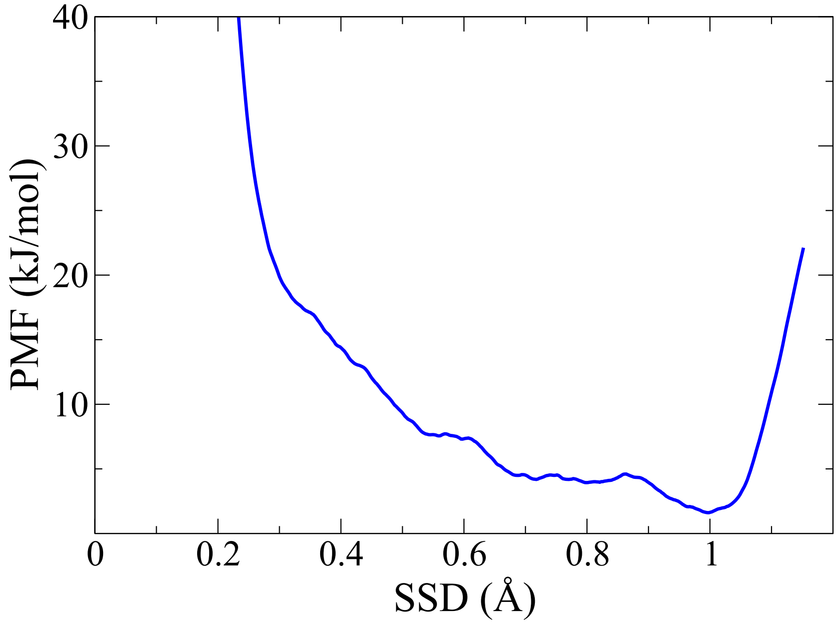


Figure S2: (Top) The SSD collective variable plotted as function of time for each walker along the Metadynamics simulation for Glycine in its negative charged state. (Bottom) The resulting PMF projected on the SSD collective variable. It is evident that Gly cannot adsorb on the $TiO_2$ anatase (101) surface due to the absence of the positive charged group $NH_3^+$ and the weak interaction of the negative charged group $COO^-$.

## S3. Nyquist plots for Glycine

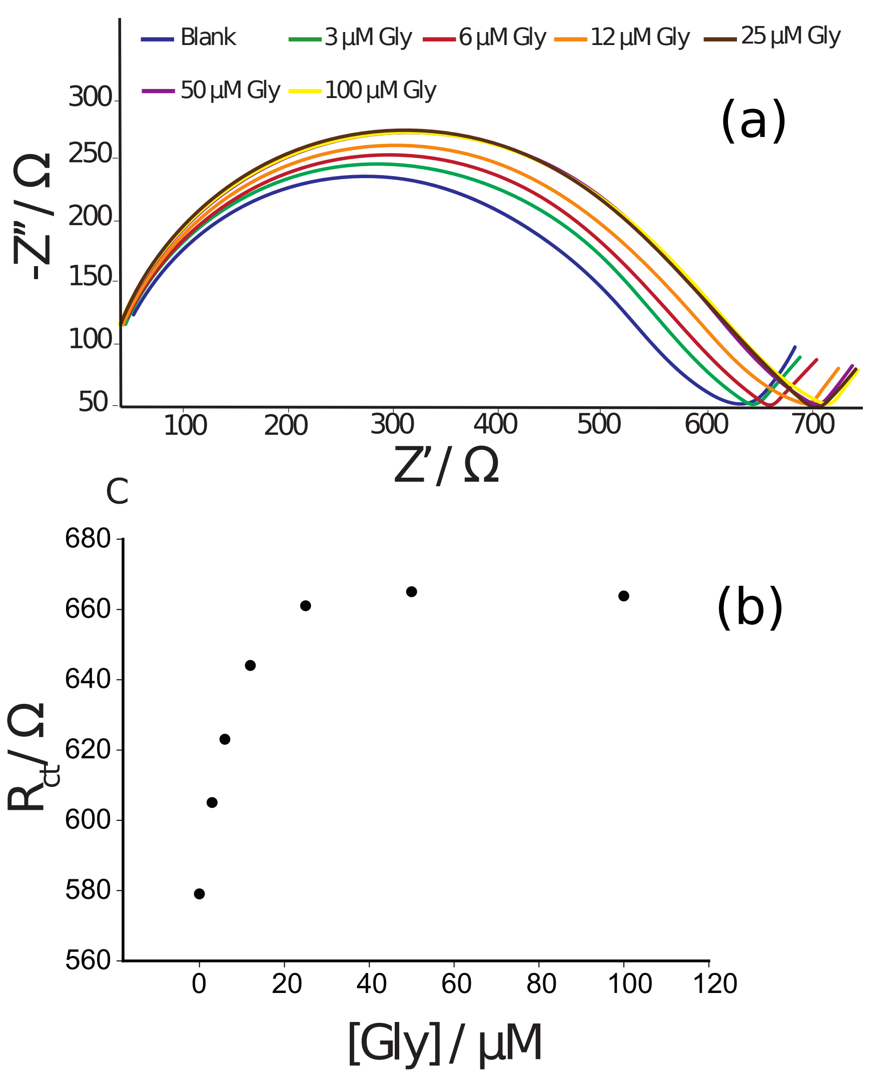


Figure S3: Nyquist plots (a) recorded in 5 mM ferro-ferrycyanide for Gly adsorbtion and the corresponding $R_{ct}$ values for Gly adsorbtion (b) on the $TiO_2$-modified gold screen-printed electrode. The Zview software was used for recording and fitting the $R_{ct}$ values, taking into account a single semicircle approximation. The fittings exclude the diffusional part of the Nyquist plot. We note that the adsorption of glycine on $TiO_2$ was studied by applying EIS measurements in ferro-ferricyanide after the adsorption process. After glycine adsorption, an increasing $R_{ct}$ value is obtained (Fig. S3), ascribable to the increment of the steric hindrance on the $TiO_2$ nanoparticles surface upon glycine layer formation.

# References


(1) Elstner, M.; Porezag, D.; Jungnickel, G.; Elsner, J.; Haugk, M.; Frauenheim, T.; Suhai, S.; Seifert, G. Self-consistent-charge density-functional tight-binding method for simulations of complex materials properties. *Phys. Rev. B* **1998**, *58*, 7260–7268.

(2) Luschtinetz, R.; Frenzel, J.; Milek, T.; Seifert, G. Adsorption of Phosphonic Acid At the $TIO_2$ Anatase (101) and Rutile (110) Surfaces. *J. Phys. Chem. C* **2009**, *113*, 5730–5740.

(3) Becke, A. D. Density-Functional Exchange-Energy Approximation With Correct Asymptotic Behavior. *Phys. Rev. A* **1988**, *38*, 3098–3100.

(4) Lee, C.; Yang, W.; Parr, R. G. Development of the Colle-Salvetti Correlation-Energy Formula Into a Functional of the Electron Density. *Phys. Rev. B* **1988**, *37*, 785–789.

(5) Grimme, S. Semiempirical GGA-Type Density Functional Constructed With a Long-Range Dispersion Correction. *Journal of Computational Chemistry* **2006**, *27*, 1787–1799.

(6) Hutter, J.; Iannuzzi, M.; Schiffmann, F.; VandeVondele, J. CP2K: Atomistic Simulations of Condensed Matter Systems. *Wiley Interdisciplinary Reviews: Computational Molecular Science* **2014**, *4*, 15–25.

(7) Tribello, G.; Bonomi, M.; Branduardi, D.; Camilloni, C.; Bussi, G. PLUMED2: New feathers for an old bird. *Comp. Phys. Comm.* **2014**, *185*.

(8) Martínez, L.; Andrade, R.; Birgin, E. G.; Martínez, J. M. Packmol: A package for building initial configurations for molecular dynamics simulations. *Journal of Computational Chemistry* **2009**, *30(13)*, 2157–2164.

(9) Agosta, L.; Brandt, E. G.; Lyubartsev, A. P. Diffusion and reaction pathways of water near fully hydrated TiO2 surfaces from ab initio molecular dynamics. *The Journal of Chemical Physics* **2017**, *147*, 024704.

(10) Bussi, G.; Donadio, D.; Parrinello, M. Canonical Sampling Through Velocity Rescaling. *The Journal of Chemical Physics* **2007**, *126*, 014101.

(11) Barducci, A.; Bussi, G.; Parrinello, M. Well-Tempered Metadynamics: A Smoothly Converging and Tunable Free-Energy Method. *Phys. Rev. Lett.* **2008**, *100*, 020603.

(12) Valsson, O.; Tiwary, P.; Parrinello, M. Enhancing Important Fluctuations: Rare Events and Metadynamics from a Conceptual Viewpoint. *Annual Review of Physical Chemistry* **2016**, *67*, 159–184.

(13) Branduardi, D.; Bussi, G.; Parrinello, M. Metadynamics with Adaptive Gaussians. *Journal of Chemical Theory and Computation* **2012**, *8*, 2247–2254.

(14) Agosta, L.; Brandt, E.; Lyubartsev, A. Improved Sampling in Ab Initio Free Energy Calculations of Biomolecules at Solid–Liquid Interfaces: Tight-Binding Assessment of Charged Amino Acids on $TiO_2$ Anatase (101). *Computation* **2020**, *8*, 12.

(15) Raiteri, P.; Laio, A.; Gervasio, F. L.; Micheletti, C.; Parrinello, M. Efficient Reconstruction of Complex Free Energy Landscapes by Multiple Walkers Metadynamics. *The Journal of Physical Chemistry B* **2006**, *110*, 3533–3539.

(16) Shchelokov, A.; Palko, N.; Potemkin, V.; Grishina, M.; Morozov, R.; Korina, E.; Uchaev, D.; Krivtsov, I.; Bol'shakov, O. Adsorption of Native Amino Acids on Nanocrystalline TiO2: Physical Chemistry, QSPR, and Theoretical Modeling. *Langmuir* **2019**, *35*, 538–550.

(17) Agosta, L.; Rzepka, P.; Chen, J.; Slabon, A.; Gordeeva, A.; Lyubartsev, A.; Hermansson, K.; Jaworksi, A. Hydrophobic signature on $TiO_2$ nanoparticles in liquid water. *ChemRxiv* **2022**,

(18) Chang, B.-Y.; Park, S.-M. Electrochemical Impedance Spectroscopy. *Annual Review of Analytical Chemistry* **2010**, *3*, 207–229.

(19) Peer Reviewed: Electrochemical Impedance Spectroscopy for Better Electrochemical Measurements. *Analytical Chemistry* **2003**, *75*, 455 A–461 A.
